# Genetic and microbiomics approaches allow the monitoring of the growth of *Dermatophagoides pteronyssinus* cultures and their environmental influences

**DOI:** 10.1371/journal.pone.0359777

**Published:** 2026-10-05

**Authors:** David Calzada, Laura Martín-López, Jerónimo Carnés

**Affiliations:** R&D Unit, Allergy and Immunology, LETI Pharma S.L.U, Madrid, Spain; Osmania University, INDIA

## Abstract

*Dermatophagoides pteronyssinus* is cultured in industrial facilities to produce allergen extracts for allergy diagnosis and therapeutic treatment. In these facilities, mite growth and production should be monitored, and exhaustive quality control is mandatory to harvest mites, reach optimal expansion, and avoid potential microbial contamination. In this study, we explored genetic approaches to monitor the growth of five independent *D. pteronyssinus* cultures. Microbiological studies were performed to characterise the evolution of microbial communities during culture. Finally, we designed a qRT-PCR application to quantify mite populations in the cultures. Our microbiome studies revealed the presence of non-pathogenic bacteria and the absence of Gram-negative bacteria. Despite the variability in microbiome genera at the beginning of the five cultures, the microbiome composition tended to be more homogeneous among the culture batches as mite growth progressed. Specifically, *Staphylococcus* sp., *Virgibacillus* sp., and *Malassezia* sp. appeared to be the most significant taxa involved in culture progression. In summary, we developed a specific method for quantifying and monitoring mite cultures, which could be used to establish an objective method for harvesting mites to manufacture standardised allergen extracts. Additionally, we provide a comprehensive description of the relationship between mites and their symbiotic microorganisms.

## Introduction

*Dermatophagoides pteronyssinus* is the predominant allergenic mite worldwide [1,2]. It produces multiple allergens that cause rhinoconjunctivitis, asthma and contribute to allergic skin diseases in genetically predisposed individuals [3]. Allergen immunotherapy (AIT) is currently the only aetiopathological treatment for allergic diseases. It involves the repeated administration of increasing amounts of allergen extracts, followed by a maintenance phase, to achieve a form of allergen-specific tolerance that provides clinical benefit for years even after discontinuation [4–6].

Allergen extracts are produced from natural sources, such as pollen or animal epithelia. Mites are mass-cultured in specific facilities of pharmaceutical companies following good manufacturing practices [7,8]. Mite cultures have a typical growth pattern. The initial phase is characterised by a latency period followed by an exponential growth period, resulting in the maximum population of mites. Determining the exact duration of mite production is critical [8,9]; this has led to the determination of the most suitable allergenic composition in the final mite allergen extract. Decisions regarding this are usually made by a qualified workforce that monitors mite growth during culture [7]. However, having an objective method to quantify the number of individuals could be an important competitive advantage, saving time and improving manufacturing yield. In this sense, quantitative real-time PCR (qRT-PCR) has been demonstrated to be a useful tool to quantify microorganisms in environmental samples [10–12]. Moreover, it has been used to identify different mite species in house dust. However, to our knowledge, this method has never been used to monitor mite production.

Mite cultures are produced in a controlled atmosphere to guarantee optimum yield because of their extreme sensitivity to environmental conditions, such as temperature, relative humidity, and diet [5,8]. However, despite efforts to control all these parameters, mite growth presents some variation among batches. Temperatures higher than 30°C and relative humidity higher than 80% can accelerate mite population growth but also favour fungal growth, which may be detrimental to mite survival [8]. Similarly, Gram-negative bacteria are associated with the production of endotoxins that contaminate mite cultures [13]. Traditional studies and culture methods have only identified convective microorganisms and do not reflect the entire composition of the mite microbiome [14]. Therefore, the biological relationship between mites and their symbiotic microorganisms remains poorly understood, and the factors affecting the dynamics of microbial communities have not been completely elucidated [15]. In recent years, the rapid evolution of next-generation sequencing methods has allowed the study of the microbiome composition in different organisms, including mite species [16–19]. Several studies have analysed the microbiome of house dust mites, while others have analysed microbial community dynamics during mite growth; however, these studies mainly focused on lab cultures and not industrial production.

Considering the relevance of mite cultures as raw materials for producing allergen extracts for immunotherapy and allergy diagnosis, we evaluated the growth of five *D. pteronyssinus* cultures and monitored their microbiomes, including the bacterial and fungal taxa. Therefore, this study focused on exploring qRT-PCR as a tool for monitoring mite growth, determining the microbial communities in different culture medium (diet) batches before the cultures started, analysing the external and internal microbial profiles across mite cultures, and correlating them with mite growth.

## Materials and methods

Fig 1 summarizes the general methodology used in this study. Five independent *D. pteronyssinus* cultures from five different CM batches were used in this study. Growth evolution and monitoring of internal and external symbiotic microorganisms were performed.

**Fig 1:**
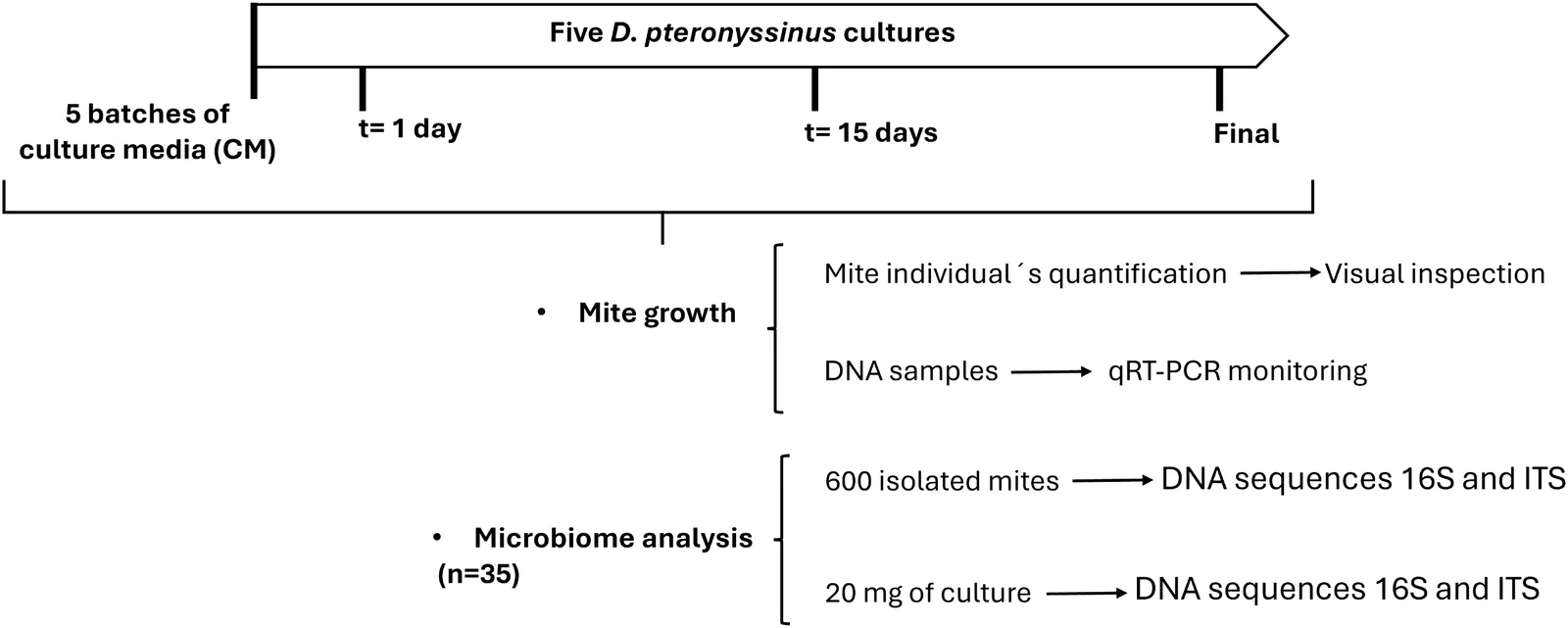
General scheme of the study.

### Establishment of *D. pteronyssinus* cultures

Five subsequentially *D. pteronyssinus* cultures (350 g per culture) were maintained at LETI Pharma facilities (Madrid, Spain) for 27–34 days. Each culture starts with 175 grams of an inoculum culture, which consists of *D. pteronyssinus* stock from LETI Pharma, and 175 grams of the culture media. The culture media used is provided by the same supplier but from five different batches. These media were previously frozen at -20°C for 48 hours. The temperature and humidity were continuously monitored and maintained throughout the culture period. The purity and richness of the mite bodies were analysed before the final harvest. Afterward, the cultures were inactivated by freezing at temperatures below -20°C for 48 h.

### Quantification of mite growth

#### Quantification of individual mites.

Five grams of the culture were sampled at different time points (days 1, 15, and the final day [days 27–34]). Briefly, 10 g of the culture was weighed and resuspended in purified water to quantify the mites. Each sample was independently counted by two trained operators, and values were averaged. The population density was defined as the number of mites per gram of the culture.

#### Quantification via qRT-PCR.

DNA isolation: Briefly, 20 µg of the *D. pteronyssinus* culture or CM was homogenised using a plastic pestle. Two independent biological replicates (DNA extractions) were performed per sample. The homogenate was extracted using a NucleoSpin Plant II kit (Macherey-Nagel, Germany) following the manufacturer’s instructions, with minor modifications. The DNA concentration and quality were determined using a spectrophotometer (ND-1000 Nanodrop; Thermo Fisher Scientific, USA). The DNA was stored at -80°C until further analysis.

DNA amplification: DNA amplification was independently performed twice per sample at two different research centers, the R&D Unit of LETI Pharma and a Genetic Unit of Centro de Biología Molecular (CBM) Severo Ochoa (CSIC-UAM) Madrid, Spain. Samples were processed in the same way. Briefly, DNA samples were diluted 1:200 and amplified using a CFX384 Real-Time System C1000 Thermal Cycler (Bio-Rad, USA). Each PCR reaction (10 μl per well) contained TaqMan™ Fast Universal PCR Master Mix, DNA samples, primers (forward: 5′-CATCCAACCAGAGTGGTATTTCC-3′ and reverse: 5′-GCTATTGCGCATACTCCACCTA-3′), and TaqMan^TM^ probe (5 ′-TATGCAATCCTTCGGGCTATCCCATCA-3′) [10]. The thermocycling conditions were as follows: 95°C for 20 s, and 40 cycles of at 95°C for 3 s and 60°C for 30 s. Each DNA amplification was analyzed in technical triplicate.

PCR data were processed using CFX Maestro software to obtain the specific Cq values for each DNA sample. This Cq value refers to cycle threshold. It represents the cycle number at which a sample’s reaction crosses a fluorescence threshold, indicating the detection of the target nucleic acid. Lower Cq values indicate higher target sequence amounts, while higher Cq values suggest lower amounts. The mean Cq value was calculated in triplicate.

### Major allergen levels

Five g of each sample from DPT cultures were extracted (1:10) in PBS 0.01M–NaCl 0.15M for 4 h at 2–8 °C, under continuous magnetic stirring. Then, samples were centrifuged at 7300*g* for 30 min, and finally the supernatants were recovered. Then, Der p 1 and Der p 2 levels were quantified by ELISA kits (Inbio Biotechnologies, Charlottesville, VA, USA) following manufacturer’s protocol.

### Microbiome analysis

Six hundred adult mites were isolated and cleaned by washing twice with 96% ethanol. To study the external microbiome, 20 mg of culture or CM alone were obtained. Samples were processed following the same methodology for DNA extraction (as described above).

Microbiome analysis was performed on a total of 35 samples corresponding to five culture batches, including internal, external, and culture medium samples collected at defined time points.

Bacterial and fungal communities were characterized by sequencing the 16S rRNA gene and the internal transcribed spacer (ITS) region, respectively, using the following primers: V3–V4 Forward: CCTACGGGNGGCWGCAG, V3-V4 Reverse: GACTACHVGGGTATCTAATCC; and ITS primers: ITS86 Forward: GTGAATCATCGAATCTTTGAA, and ITS4 Reverse: TCCTCCGCTTATTGATATGC. Sequencing was performed on a MiSeq platform (Illumina, San Diego, CA, USA) at the Genomics and NGS Core Facility of (CBM-SO). Quality analyses were performed on the reads using FastQC^2^ software. Primer sequences were removed, and chimeric sequences were discarded using PANDAseq Assembler and QIIME 2 software. Over 80% of the joined reads were recovered from all the samples. Apart from that chloroplast and mitochondria barcode sequencing contamination in 16S were removed although the number of sequences assigned to them was quite low, with maximum values of 0.6% (mitochondria) and 3.5% (chloroplast). Regarding potential mite ITS contamination, the UNITE database is specifically curated for fungal ITS sequences and does not comprehensively represent metazoan taxa. A detailed bioinformatic workflow is included in the S1 File (Supporting Information). The raw data were deposited in the European Nucleotide Archive (accession number: PRJEB80324). Alignment, phylogenetic tree construction, taxonomic assignment analyses, and functional profiling were performed using two different platforms: https://www.microbiomeanalyst.ca/ and STAMP (Statistical Analysis of Metagenomic Profiles) [20,21].

### Statistical analysis

The mite population and Cq data were analysed using GraphPad Prism 9 software. Pearson correlations between Cq and mite density (mites/g) were computed per culture and globally; significance threshold p < 0.05. Finally, to evaluate the effects of time and culture conditions on mite population and Cq, two-way ANOVA was performed.

For microbiome data, beta diversity was assessed using principal coordinate analysis (PCoA) and hierarchical clustering based on Bray–Curtis distance matrices computed at the ASV (feature) level from data exported from QIIME2. Differences in microbial community composition between groups were evaluated using PERMANOVA, and p-values were calculated using permutation tests (999 permutations), as implemented in MicrobiomeAnalyst.

For taxonomic comparisons differences in relative abundances of taxon-levels between sample groups were evaluated using STAMP (v2.1.3) which allows statistical comparison of metagenomic profiles using multiple hypothesis testing and effect size estimation. Comparisons were conducted to identify taxa showing significant differences between samples and culture conditions on relative abundance data at the genus level. Differences between groups were assessed using the G-test (with Yates’ correction) and Fisher’s exact test when appropriate. Multiple testing correction was applied using Bonferroni correction. Taxa with adjusted p < 0.05 were considered statistically significant.

## Results

### *D. pteronyssinus* growth

Mite cultures were grown in the absence of contaminants for the entire experimental period. Regarding the mite populations, Fig 2A shows the growth curve of each culture obtained via visual inspection. Culture 1 had a fast and persistent evolution and finished with the highest concentration of mites (181,783 mites/g culture), followed by culture 2 with a final concentration of 146,226 mites/g culture. Culture 5 grew rapidly during the first 15 days and then slowly during the last phase of culture. Cultures 3 and 4 grew slowly and had the lowest mite levels (99,434 and 106,865 mites/g of culture, respectively).

**Fig 2:**
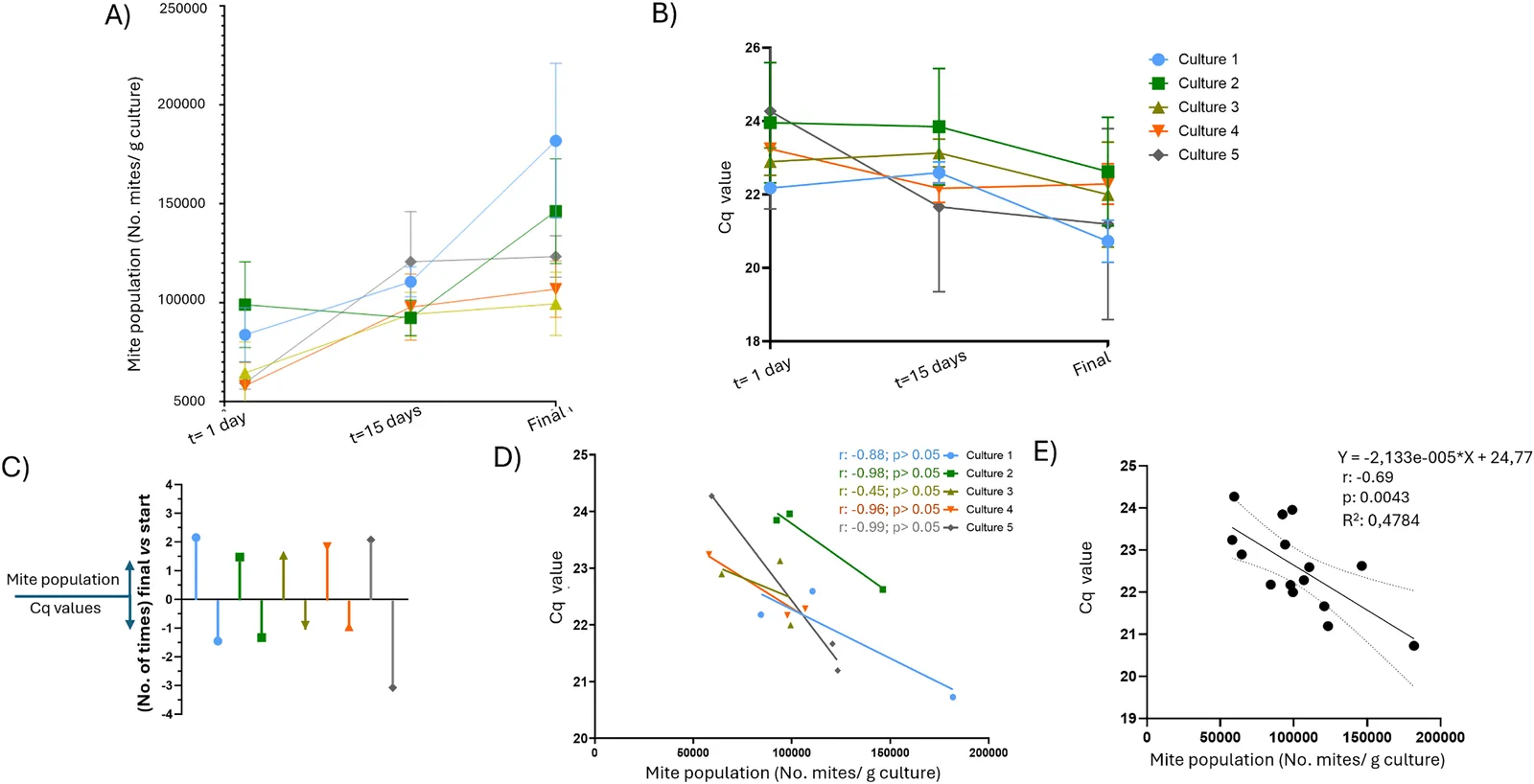
Mite growth. (A) Dynamics of the mite population (average mite number ± standard deviation) is shown (B) Mite DNA quantification reported by qRT-PCR in terms of Cq evolution during mite culture (average Cq ± standard deviation). (C) Changes in the mite population and Cq between the final stage and the beginning of the mite cultures. (D) Correlation studies between the mite population and Cq in each culture. (E) Global correlation study.

The two-way ANOVA showed a significant effect of time on mite population (F(2,8) = 11.66, p = 0.0043), accounting for a large proportion of the total variance (57.5%), indicating a consistent increase over time. These results are consistent with the trends observed in Fig 2D-E.

In contrast, no significant differences were observed between culture types (F(4,8) = 2.308, p = 0.1460), suggesting that mite population dynamics are primarily driven by temporal factors rather than culture conditions.

According to the qRT-PCR results (Fig 2B), culture 1 had the lowest Cq value, with a reduction of 1.45 cycles from the beginning, while culture 5 had the highest change Cq value (3.07). Samples from the final step of cultures 3 and 4 also had low values, at 0.90 and 0.95, respectively (Fig 2C). Considering this data, we observed that an increase in population doubling was associated with a reduction of more than 1.45 cycles (Fig 2D). In this respect, the correlation between the Cq values and mite population observed was significant (r: -0.69; p-value: 0.0443) (Fig 2E).

To further investigate the relationship between Cq values and experimental factors, a two-way ANOVA was performed using Cq as the dependent variable.

The analysis showed a significant effect of both time (two-way ANOVA, F(2,8) = 5.716, p = 0.0287) and culture type (F(4,8) = 5.485, p = 0.0200), indicating that Cq values are influenced not only by temporal changes but also by culture-specific conditions.

Regarding allergen levels quantified along the mite cultures, Culture 1 and 4 ended with the highest levels of Der p 1 while Der p 2 levels tended to be more similar at the end of the cultures (S1 Fig in S1 File).

### Microbiome diversity in *D. pteronyssinus* cultures

The raw sequences were denoised, dereplicated, and filtered; the details are included in S1File of Supporting Information. Briefly, the total numbers of non-chimeric sequences used for analysis were 144,712–326,881 for *16S rRNA*, and 72,095–68,646 for *ITS*. The sequences of all samples were grouped to define amplicon sequence variants (ASVs), which were equivalent to 100% operational taxonomic units. We obtained a total number of 2,195 ASVs for *16S rRNA* and 917 ASVs for *ITS*. All ASV-level abundance and taxonomy data are provided in Supplementary S2 Table (16S) and in Supplementary S3 Table (ITS).

All the samples included in the study of bacterial communities belonged to three phyla: Firmicutes, Actinobacteria, and Proteobacteria. The heat tree analysis leverages the hierarchical structure of taxonomic classifications. Firmicutes were mainly derived from two large families, Staphylococcaceae and Bacillaceae. Actinobacteria were derived from the families Micrococcales and Propionibacteriaceae, while Proteobacteria were primarily derived from Burkholderiales and Enterobacteriales. The most common genera were *Staphylococcus* sp., followed by *Virgibacillus* sp., *Kocuria sp*., “Other,” “Not assigned,” *Streptomyces sp*., and *Cutibacterium* sp. (Fig 3A).

**Fig 3:**
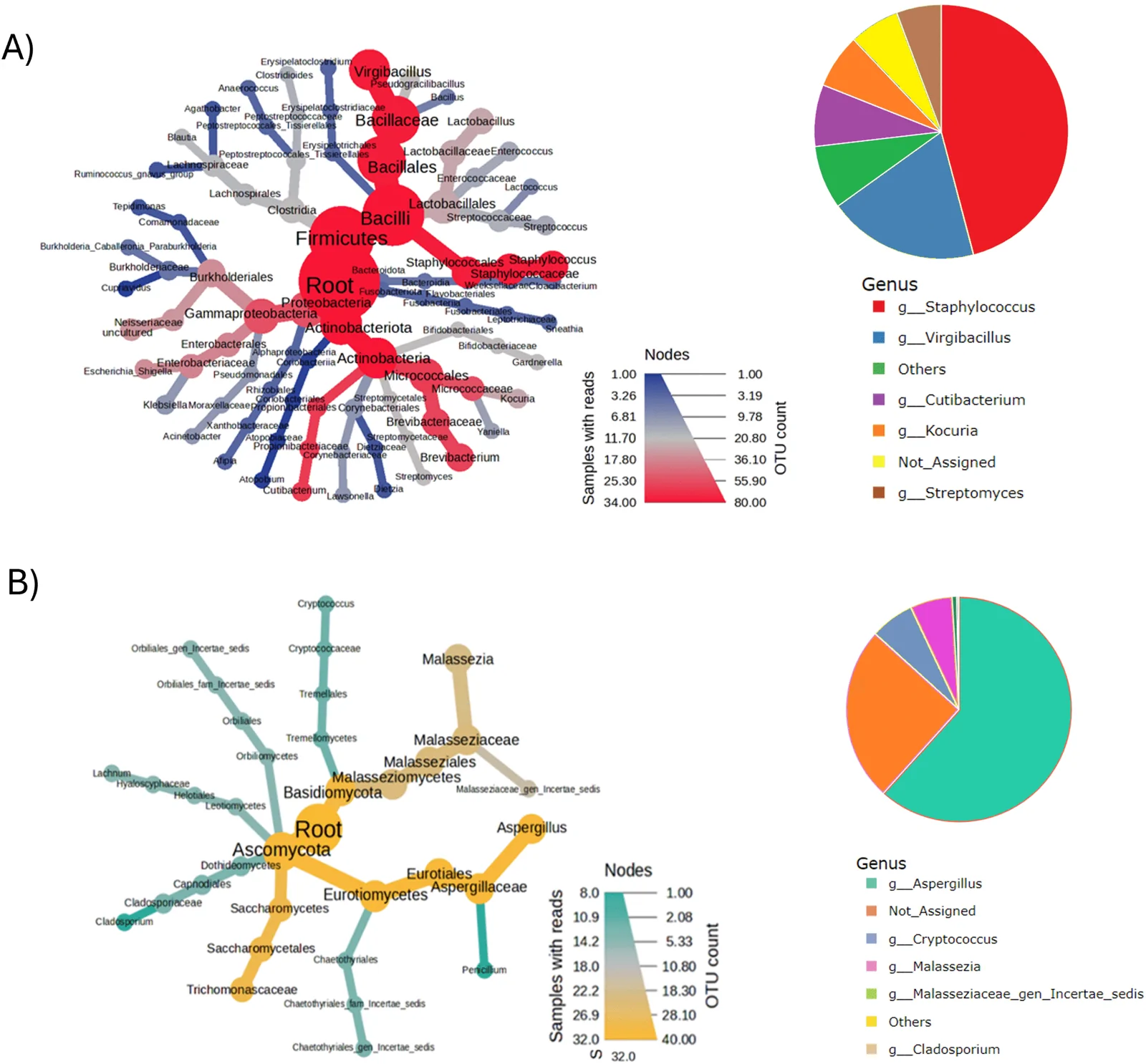
Microbiome diversity in *Dermatophagoides pteronyssinus* cultures. (A) Heat tree and pie chart of the most common bacterial genera found in all samples, without considering the culture batch or the origin of the samples (external or internal). (B) Fungi analysis: Heat tree and pie chart of the most common six fungal genera found in all samples. The “Not_Assigned” category includes sequences that could not be reliably classified at the genus level, although they are assigned to higher taxonomic ranks. “Other” category groups low-abundance genera that are individually identified but grouped for visualization purposes.

The fungal taxa are presented in Fig 3B, showing two major phyla: Ascomycota and Basidiomycota. Ascomycota were mainly derived into two large families, Saccharomycetes and Eurotiomycetes, while Basidiomycota were derived into genera *Malassezia* and *Cryptococcus*. In terms of genera, *Aspergillus sp.* was the most common, followed by *Cryptococcus sp., Malassezia sp.*, and *Cladosporium sp.*

To improve the interpretation of the global microbiome structure across samples, we performed unsupervised hierarchical clustering and principal coordinates analysis (PCoA) using all bacterial and fungal taxonomic profiles (Fig 4).

**Fig 4:**
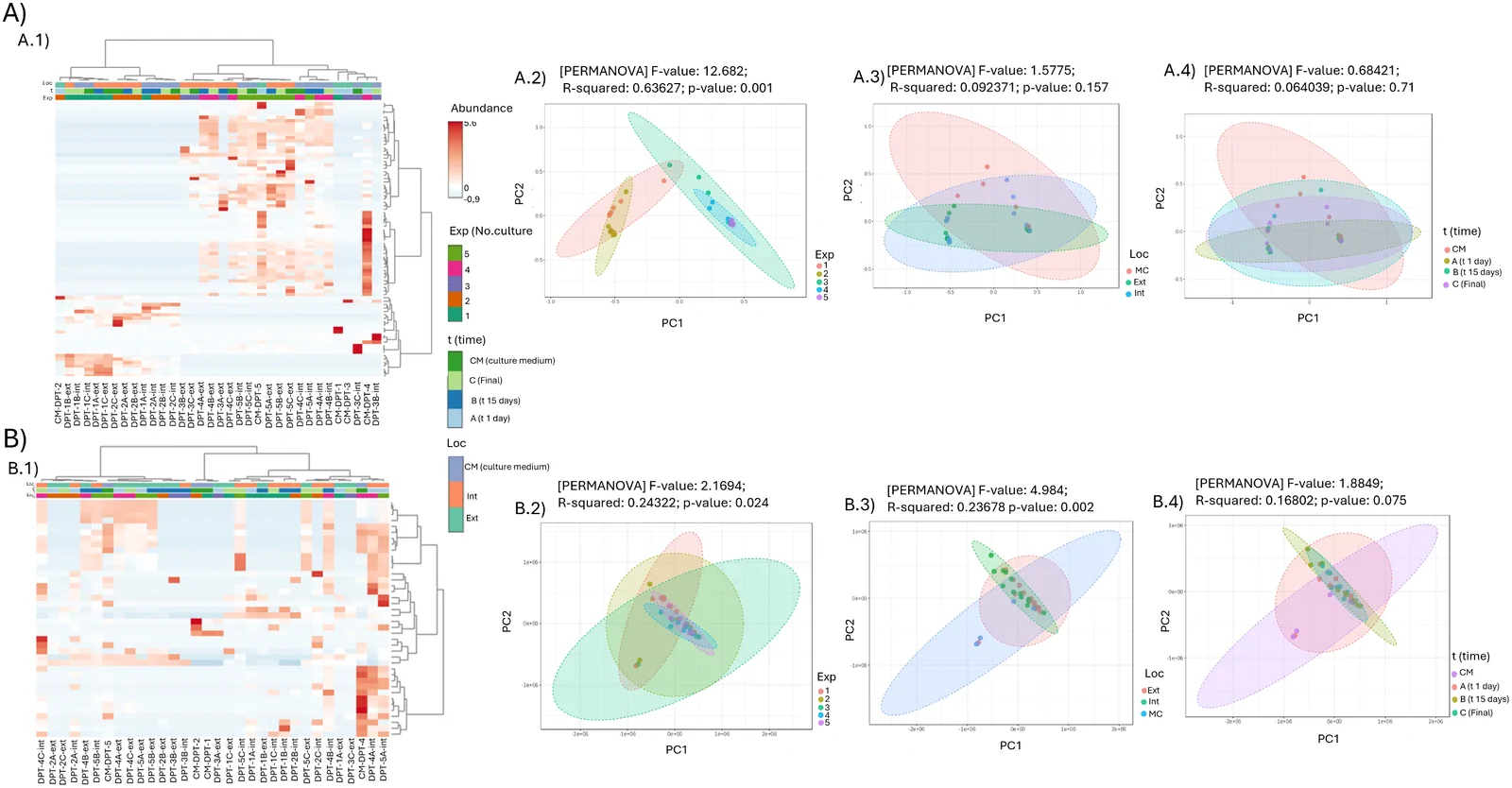
Unsupervised hierarchical clustering of all bacterial and fungal DNA samples. (A) **Bacterial taxa.** (A.1) Heatmap with hierarchical clustering identifying two major sample clusters: cultures 1–2 vs. cultures 3–5, with culture media (CM) forming a distinct branch. (A.2) PCoA grouped by culture batch confirming this separation. (A.3) PCoA grouped by sample type (CM, external, internal). (A.4) PCoA grouped by culture time. (B) **Fungal taxa.** (B.1) Heatmap showing overall homogeneous fungal profiles across samples. (B.2–B.4) PCoA plots grouped by batch, sample type, and time. Exp (No. culture batches; Loc (localization; external or internal microbiome).

For the bacterial taxa, the heatmap and clustering analysis (Fig 4A.1) revealed the presence of two major sample clusters. Cultures 1 and 2 grouped together, whereas cultures 3, 4, and 5 formed a second cluster. Samples corresponding to the CM 1, 3 and 4, before inoculation, branched separately, indicating distinct bacterial profiles at baseline. The PCoA based on culture batches (Fig 4A.2) confirmed this separation (PERMANOVA, F = 12.682, R^2^ = 0.636, p= 0.001), supporting the association between bacterial community structure and the different growth behaviors observed among cultures. In contrast, PCoA grouped by sample origin (culture medium, external, or internal microbiota; Fig 4A.3) and culture time (Fig 4A.4) did not show defined clustering patterns, indicating that these two factors did not drive the major sources of variability in bacterial composition.

For the fungal taxa, the heatmap (Fig 4B.1) showed more homogeneous mycobiome profiles across all samples in comparison with bacteria. Likewise, PCoA plots grouped by culture batch and sample origin display moderate clustering according to any of these variables (PERMANOVA, F = 2.169, R^2^ = 0.243, p = 0.024; and F = 4.984, R^2^ = 0.237, p = 0.002, respectively) but not in the case of time (Fig 4B.2–4B.4). These results indicate that the fungal community remained relatively stable across developmental stages.

### Microorganisms in culture media batches

Considering only the samples from the culture media (CM) batches before the cultures started, it was observed that they did not have conserved genera in terms of relative bacterial abundance. *Cutibacterium sp.* and “other genera” were the most common taxa present in the CM, followed by *Staphylococcus sp.* and *Virgibacillus sp.* (Fig 5A.1). CM3 and CM5 had the highest percentages of *Cutibacterium sp.* and *Staphylococcus sp*., respectively. The presence of these genera was statistically significant compared to that in the other four cultures. Among the fungal taxa, *Criptococcus sp.* was the most abundant in CM1 and CM2. *Malassezia sp.* was the most common in CM4, while *Aspergillus sp.* was the most common in CM5. Statistically significant differences were observed among the CM batches, as shown in Fig 5A.2.

**Fig 5:**
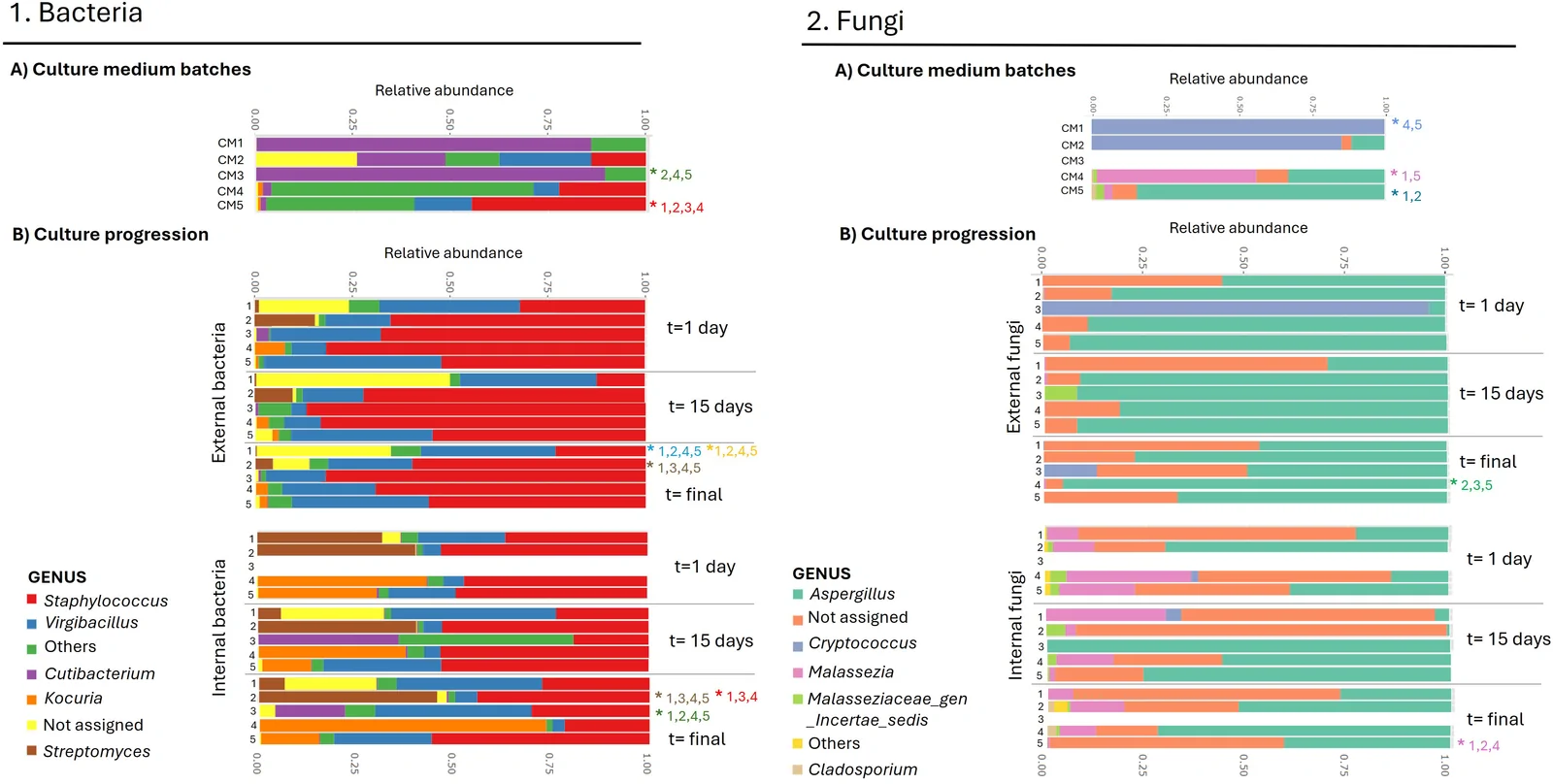
Microbiome profiles in terms of relative abundance. (A) Culture medium (CM) batches and (B) culture development time. Left graphs: bacteria diversity. Right panel: fungal diversity. Statistically significant differences among cultures at the same time of the study are denoted (previous culture development and final stage) as *, with p values < 0.05. “Not-Assigned” group refers to those that could not be reliably classified at the genus level but are assigned to higher taxonomic ranks (e.g., phylum, class or order). “Other” category groups low-abundance genera that are individually identified but grouped for visualization purposes.

### Changes in microbial communities during mite culture growth

Among the five cultures, the external microbiome was more homogeneous than the internal microbiome and tended to be similar among batches. The most common bacterial genera in the external environment were *Staphylococcus sp.* and *Virgibacillus sp*. In the case of internal bacteria, apart from both genera, *Kocuria sp.* and *Streptomyces sp*. appeared as the other relevant bacterial genera (Fig 5B.1). Considering the fungal taxa, *Asperigillus sp.*, an unassigned group, and *Cryptococcus sp.* were the most abundant in the external samples; however, *Malassezia sp* was found to be another relevant genus in the internal samples.

A comparison of the evolution of the five cultures revealed several variations in the relative abundance of the bacterial genera from the beginning to the end of the culture. Bacteria from the external samples were similar across batches at the end of the culture. In contrast, internal bacteria showed major changes during the growth of the culture, depending on the culture batch used. Cultures 4 and 5 had similar internal bacterial diversity patterns at the end of the cultures. Culture 2 had the highest abundances of *Streptomyces sp.* and *Staphylococcus sp.* (STAMP analysis; G-test with Yates’ correction, Bonferroni-adjusted p < 0.05 compared with the other four cultures), and culture 3 was the only culture that presented *Cutibacterium sp.* with statistically significant differences (Fig 5B.1). Regarding the fungal abundance (Fig 5B.2), only culture 3 presented *Cryptococcus sp.* in the final stage of the external samples. The remaining cultures ended only with *Aspergillus sp.* and were not assigned to any genera. *Aspergillus sp.* was more common in culture 4 than in other cultures (STAMP analysis; G-test with Yates’ correction, Bonferroni-adjusted p < 0.05). Regarding internal fungi, only culture 5 did not present *Malassezia sp.* at the end of the culture (culture 3 could not be analysed).

### Relationship between microbial communities and mite growth

Cultures 4 and 5 showed similar evolution in terms of growth patterns and shared the same bacterial taxa in both external and internal samples. With respect to the other cultures, culture 1 had the highest rate of mite growth and presented the highest relative abundance of “Not assigned” and *Virgibacillus* taxa in the external bacteria, followed by culture 2 with the highest abundance of *Streptomyces* in the internal and external samples and *Staphylococcus sp*. in the external samples. In contrast, culture 3 had the lowest mite growth and was the only culture that contained *Cutibacterium sp.* in the internal microbiome and *Cryptococcus sp.* in the external microbiome.

## Discussion

Growing mites require the maintenance of cultures under proper conditions to guarantee and harmonise the raw materials used and produce the desired allergen extracts [8,9]. Therefore, the quality of an allergen extract is related to the adequacy of the original culture and its final stage. Moreover, mite production is a critical procedure that requires specialised knowledge, is time-consuming, and presents high costs to the industry [7]. In contrast, straightforward methods based on molecular markers can be used to quantify the progress of a culture. Thus, qRT-PCR could be an optimal methodology and a cost-effective alternative for evaluating mite production. In fact, the usefulness of qRT-PCR to quantify house dust mites and storage mites in the dwellings of patients allergic to mites has already been demonstrated [10]

Our results suggest that this technology successfully monitored the progress of five *D. pteronyssinus* cultures in this study. Regarding the Cq values, the profiles of the five cultures were broadly consistent with mite growth behaviour. The correlation values (r) between the Cq values and mite populations in cultures 1, 2, 4, and 5 ranged from -0.88 to -0.99. Culture 3 was the only one with an r value less than -0.5, likely due to its reduced growth rate and lower final mite population.

To further explore these observations, we performed a two-way ANOVA using Cq as the dependent variable. In contrast to direct mite counts, Cq values were significantly influenced by both time and culture type, indicating that, while Cq reflects overall temporal trends in mite growth, it is also affected by culture-specific factors.

This additional variability likely reflects the indirect nature of qRT-PCR measurements, which can be influenced by DNA extraction efficiency, sample composition, or amplification variability. Therefore, qRT-PCR should be considered a reliable and practical tool for monitoring mite cultures, but complementary to direct population measurements rather than a direct equivalent.

Overall, our results support the use of this genetic approach for process monitoring and potential certification in mite production, reducing time requirements and subjective interpretation, and improving production yield. However, further studies including a larger number of samples will be necessary before full implementation.

On the other hand, the mite microbiota is closely related to the progress of culture [14,15,19]. In this study, we investigated the temporal variations in the microbial composition of five industrial mite cultures. Regarding the diet, we know that one limitation of this study is that we could not indicate exactly which is the exactly composition of media culture, because it is confidential but here were several differences among culture media batches. Culture media 1, 2, and 3 showed more differences in bacterial and fungal taxa compared to batches 4 and 5. However, the microbiome composition tended to be more homogeneous among the culture batches as mite growth progressed. Therefore, studying the microbiome composition of the diet before the start of culture could be crucial to eliminate potential variations, and this should be analysed in detail. It should be mentioned that despite mite cultures growing with the presence of all these symbiotic microorganisms, the entire manufacturing process to obtain final allergen extracts includes a final filtration step through a 0.2-mM filter renders the extract sterile.

Bacterial analysis showed that *D. pteronyssinus* cultures contained non-pathogenic bacteria with a few dominant taxa. Only three main phyla were observed: Firmicutes, Actinobacteria, and Proteobacteria, in line with other related studies that used environmental samples and laboratory cultures [14,22]. Specifically, *Staphylococcus sp.*, *Virgibacillus sp.* and *Kocuria sp.* were the most common genera, as previously demonstrated in *D. pteronyssinus* and *D. farinae* cultures [15,19,23]. Interestingly, a non-significant proportion of Gram-negative bacteria was observed in our cultures, which is a potential concern as they can produce endotoxins that may affect the production of allergen extracts [19]. These results are consistent with those of our group, where the absence of Gram-negative bacteria and endotoxins was previously reported [9]. The differences in the bacterial profiles of the five cultures could explain the differences in their growth profiles.

Regarding the principal coordinates analysis (PCoA) analysis, cultures 1 and 2, which had the highest number of individual mites at the final stage, showed the most significant differences in external and internal bacterial taxa compared to the other three cultures. These differences were attributed to the proportions of *Virgibacillus sp.,* “not assigned” taxa, *Staphylococcus sp.,* and *Streptomyces sp*. The presence of *Staphylococcus sp.* was previously shown to be positively correlated with mite density in environmental samples [19]. In contrast, the presence of *Virgibacillus sp.* in internal samples was negatively associated with mite density in mite cultures [9,19]. *Cutibacterium sp.* was observed only in the internal samples of culture 3; to our knowledge, this is the first time it has been observed in mite cultures.

Regarding fungal taxa, non-significant differences were observed among culture batches. Moreover, samples were not clustered according to this criterion, nor were there differences among the batch number, time of culture, or origin of the samples (internal or external samples). *Aspergillus sp.* was the fungal genus with the highest abundance in *D. pteronyssinus* cultures. The presence of this genus is associated with mature cultures and an increase in the culture age [15,19,23]. *Malassezia sp.* was also present in the internal mite community, as observed in similar studies, due to its persistent symbiotic association with *D. pteronyssinus* [19].

According to Der p 1 and Der p 2 results, levels of Der p 2 and microbiome composition tended to be more homogeneous among the culture batches as mite growth progressed. In the case of Der p 1, cultures 1 and 4 finished the culture with the highest levels of this major allergen, both showed the lowest levels of *Staphylococcus* and *Malasezzia* genera in internal microbiota. Despite these interesting results obtained in bacterial and fungal profiles, more studies on how commensal gut microbial communities affect the expression of mite allergens during culture are needed, particularly in the case of major allergens, Der p 1, Der p 2, and Der p 23, which are abundant in the mite gut and faecal pellets [4].

In summary, we demonstrated the utility of qRT-PCR as a promising tool for monitoring mite growth that could be used as a control mechanism to improve and optimise industrial cultures in the future. Additionally, we described variations in the microbiota of *D. pteronyssinus* cultures as environmental factors that affect the growth, development, and reproduction of mites. *Staphylococcus sp., Virgibacilluss sp*., and *Malassezia sp.* were the most abundant taxa associated with culture progression. The discovery of these non-pathogenic microorganisms is relevant from a medical perspective, considering that these mite cultures are the raw materials used to produce allergen extracts for allergy diagnosis and therapy, leading to deeper knowledge and control of mite growth. One practical application of this research could be the implementation of standard screening methods to select the best culture medium batches and monitor mite growth based on the symbiotic microorganisms present.

## Supporting information

S1 File Provides the methodologic information of the microbiome diversity workflow and results. It includes the read set tables and S1 Fig related to major allergen quantification.
(DOCX)

S2 Table ASV-level bacterial abundance and taxonomy table generated from 16S rRNA sequencing. An explicit filtering step to remove chloroplast- and mitochondrial-derived reads was performed. The table includes all amplicon sequence variants (ASVs) detected across internal, external and culture medium samples from the five *D. pteronyssinus* cultures. For each ASV, the table reports the full taxonomic assignment (from domain to genus), total abundance across all samples, prevalence (number of samples in which the ASV was detected), and read counts for every individual sample.
(XLSX)

S3 Table OTU/ASV table for the ITS (fungal) dataset showing sample-level abundances, taxonomic assignment and summary metrics. The table includes OTU ID, full taxonomic classification, total abundance across all samples, prevalence (number of samples in which the OTU is detected), and raw abundance values for each sample.
(XLSX)
