# Supplementary material for: Genetic and microbiomics approaches allow the monitoring of the growth of Dermatophagoides pteronyssinus cultures and their environmental influences
Source: PLoS One. 2026 Oct 5;21(10):e0359777. doi: 10.1371/journal.pone.0359777 (PMC13637924; doi:10.1371/journal.pone.0359777)
Supplement: Supplementary file 1 [file pone.0359777.s001.docx]

**Supporting Information**

**Material and Methods**

**Microbiome diversity in *D. pteronyssinus* cultures**

Metagenomics experiment to determine mite culture microorganisms (prokaryote and fungi) from 5 different cultures at 3 different timepoints and 2 different locations (internal and external) S1 Table 1 indicates the sample characteristics (N=35). S1 Table 2 indicates the primers sequences used.

**S1 Table 1. Sample characteristics**

| Group | Experiment | Timepoint | Location |
| --- | --- | --- | --- |
| DPT-1A-ext | 1 | A - initial | Ext |
| DPT-1A-int | 1 | A - initial | Int |
| DPT-1B-ext | 1 | B - intermediate | Ext |
| DPT-1B-int | 1 | B - intermediate | Int |
| DPT-1C-ext | 1 | C - end | Ext |
| DPT-1C-int | 1 | C - end | Int |
| DPT-2A-ext | 2 | A - initial | Ext |
| DPT-2A-int | 2 | A - initial | Int |
| DPT-2B-ext | 2 | B - intermediate | Ext |
| DPT-2B-int | 2 | B - intermediate | Int |
| DPT-2C-ext | 2 | C - end | Ext |
| DPT-2C-int | 2 | C - end | Int |
| DPT-3A-ext | 3 | A - initial | Ext |
| DPT-3A-int | 3 | A - initial | Int |
| DPT-3B-ext | 3 | B - intermediate | Ext |
| DPT-3B-int | 3 | B - intermediate | Int |
| DPT-3C-ext | 3 | C - end | Ext |
| DPT-3C-int | 3 | C - end | Int |
| DPT-4A-ext | 4 | A - initial | Ext |
| DPT-4A-int | 4 | A - initial | Int |
| DPT-4B-ext | 4 | B - intermediate | Ext |
| DPT-4B-int | 4 | B - intermediate | Int |
| DPT-4C-ext | 4 | C - end | Ext |
| DPT-4C-int | 4 | C - end | Int |
| DPT-5A-ext | 5 | A - initial | Ext |
| DPT-5A-int | 5 | A - initial | Int |
| DPT-5B-ext | 5 | B - intermediate | Ext |
| DPT-5B-int | 5 | B - intermediate | Int |
| DPT-5C-ext | 5 | C - end | Ext |
| DPT-5C-int | 5 | C - end | Int |
| MC-DPT-1 | 1 | 0 | Culture medium |
| MC-DPT-2 | 2 | 0 | Culture medium |
| MC-DPT-3 | 3 | 0 | Culture medium |
| MC-DPT-4 | 4 | 0 | Culture medium |
| MC-DPT-5 | 5 | 0 | Culture medium |
|  |  |  |  |

**S2 Table 2. Primer sequences**

| Primer name | Sequence |
| --- | --- |
| 16S V3-V4 FW | CCTACGGGNGGCWGCAG |
| 16S V3-V4 RV | GACTACHVGGGTATCTAATCC |
| ITS86F | GTGAATCATCGAATCTTTGAA |
| ITS4R | TCCTCCGCTTATTGATATGC |

Raw paired-end reads (Illumina MiSeq, 2×300 bp) from both 16S (V3–V4) and ITS amplicons underwent initial quality assessment using FastQC. Primer sequences were removed with Cutadapt (error rate 0.1, minimum overlap 3 bp). For 16S, forward and reverse reads were subsequently merged using PANDAseq (minimum overlap 100 bp, maximum 500 bp). Due to low-quality 3' ends, ITS reads were trimmed to a maximum length of 200 nt prior to downstream analysis.

Amplicon Sequence Variants (ASVs) were generated using Qiime2. Reads were imported as Qiime2 artifacts (.qza), and denoising, dereplication and chimera filtering were performed with the q2‑dada2 plugin. For 16S, the denoise-single workflow was used on already‑merged reads; for ITS, paired-end denoising (denoise‑paired) was applied, using a maximum expected error threshold of 3. The resulting feature tables and representative sequences produced 2,195 ASVs for 16S and 917 ASVs for ITS.

Taxonomic assignment of ASVs was performed using SILVA release 138 (99% identity) for bacterial 16S sequences and UNITE v9.0 (99% identity) for fungal ITS sequences. Assignments were refined by exporting BLAST results and retaining only top‑bitscore hits before re-importing the filtered taxonomy.

Representative sequences were aligned with MAFFT, filtered, and used to construct phylogenetic trees with FastTree, followed by midpoint rooting. Alpha- and beta‑diversity metrics were computed using Qiime2’s core‑metrics‑phylogenetic pipeline, including Faith’s phylogenetic diversity, Shannon diversity, observed ASVs, Bray–Curtis, Jaccard, weighted and unweighted UniFrac distances, with PCoA visualized via EMPeror.

All analyses were performed independently for 16S and ITS datasets using the structured workflow described above.

Alignment, phylogenetic tree construction, taxonomic assignment analyses, and functional profiling were performed using two different platforms: https://www.microbiomeanalyst.ca/ and STAMP.

Regarding the main abundance of bacteria and fungal genera heat tree analysis and pie chart were performed after data normalization in <https://www.microbiomeanalyst.ca/> exported from QIIME2.

Beta diversity profiling was studied by PCoA and hierarchical clustering were computed on relative abundance matrices at the feature- levels. Distance method used was Bray-Curtis Index and P ERMANOVA was used to assess differences between groups, with p-values calculated using permutation tests (999 permutations). Finally, the relative abundance were analyzed at genera levels including the top 6. These data were represented as stacked bar plots to summarize relative abundance patterns across samples. Comparisons were assessed using the G-test (with Yates’ correction) and Fisher’s exact test when appropriate. Multiple testing correction was applied using Bonferroni correction. Taxa with adjusted p < 0.05 were considered statistically significant.

**Results**

**Major allergen levels**

**S1 Fig 1. Major allergen levels in *D. pteronyssinus* cultures at day 1, 15 and at the end of the cultures.**

**Microbiome diversity in *D. pteronyssinus* cultures**

**Reads sets:** Original data include 140 read files in FASTQ format. The main characteristics of the reads are summarized in S1Table 3.

**S1 Table 3. General characteristics of reads.**

| Sample | #Reads | Group | Type of reads |
| --- | --- | --- | --- |
| MC-DPT-1_16S | 171,327 | M | 2x300 |
| DPT-1A-int_16S | 170,226 | A-int | 2x300 |
| DPT-1B-int_16S | 191,201 | B-int | 2x300 |
| DPT-1C-int_16S | 162,187 | C-int | 2x300 |
| DPT-1A-ext_16S | 188,342 | A-ext | 2x300 |
| DPT-1B-ext-_16S | 189,671 | B-ext | 2x300 |
| DPT-1C-ext_16S | 222,090 | C-ext | 2x300 |
| MC-DPT-2_16S | 172,893 | M | 2x300 |
| DPT-2A-int_16S | 174,479 | A-int | 2x300 |
| DPT-2B-int_16S | 165,897 | B-int | 2x300 |
| DPT-2C-int_16S | 182,645 | C-int | 2x300 |
| DPT-2A-ext_16S | 179,261 | A-ext | 2x300 |
| DPT-2B-ext_16S | 194,960 | B-ext | 2x300 |
| DPT-2C-ext_16S | 176,492 | C-ext | 2x300 |
| DPT-3A-ext_16S | 290,553 | A-ext | 2x300 |
| DPT-3B-ext_16S | 314,944 | B-ext | 2x300 |
| DPT-3B-int_16S | 298,889 | B-int | 2x300 |
| DPT-3C-ext_16S | 322,682 | C-ext | 2x300 |
| DPT-3C-int_16S | 266,711 | C-int | 2x300 |
| DPT-4A-ext_16S | 386,297 | A-ext | 2x300 |
| DPT-4A-int_16S | 371,969 | A-int | 2x300 |
| DPT-4B-ext_16S | 360,370 | B-ext | 2x300 |
| DPT-4B-int_16S | 384,794 | B-int | 2x300 |
| DPT-4C-ext_16S | 337,867 | C-ext | 2x300 |
| DPT-4C-int_16S | 371,477 | C-int | 2x300 |
| DPT-5A-ext_16S | 382,455 | A-ext | 2x300 |
| DPT-5A-int_16S | 330,058 | A-int | 2x300 |
| DPT-5B-ext_16S | 348,749 | B-ext | 2x300 |
| DPT-5B-int_16S | 372,821 | B-int | 2x300 |
| DPT-5C-ext_16S | 367,194 | C-ext | 2x300 |
| DPT-5C-int_16S | 359,823 | C-int | 2x300 |
| MC-DPT-3_16S | 314,143 | M | 2x300 |
| MC-DPT-4_16S | 406,103 | M | 2x300 |
| MC-DPT-5_16S | 384,746 | M | 2x300 |
| MC-DPT-1_ITS | 105,998 | M | 2x300 |
| DPT-1A-int_ITS | 249,389 | A-int | 2x300 |
| DPT-1B-int_ITS | 314,303 | B-int | 2x300 |
| DPT-1C-int_ITS | 223,005 | C-int | 2x300 |
| DPT-1A-ext_ITS | 287,058 | A-ext | 2x300 |
| DPT-1B-ext-_ITS | 228,233 | B-ext | 2x300 |
| DPT-1C-ext_ITS | 269,280 | C-ext | 2x300 |
| MC-DPT-2_ITS | 143,283 | M | 2x300 |
| DPT-2A-int_ITS | 229,394 | A-int | 2x300 |
| DPT-2B-int_ITS | 256,244 | B-int | 2x300 |
| DPT-2C-int_ITS | 202,104 | C-int | 2x300 |
| DPT-2A-ext_ITS | 191,543 | A-ext | 2x300 |
| DPT-2B-ext_ITS | 235,994 | B-ext | 2x300 |
| DPT-2C-ext_ITS | 202,601 | C-ext | 2x300 |
| DPT-3A-ext_ITS | 482,719 | A-ext | 2x300 |
| DPT-3B-ext_ITS | 635,009 | B-ext | 2x300 |
| DPT-3B-int_ITS | 349,475 | B-int | 2x300 |
| DPT-3C-ext_ITS | 558,250 | C-ext | 2x300 |
| DPT-4A-ext_ITS | 599,100 | A-ext | 2x300 |
| DPT-4A-int_ITS | 564,475 | A-int | 2x300 |
| DPT-4B-ext_ITS | 635,440 | B-ext | 2x300 |
| DPT-4B-int_ITS | 521,364 | B-int | 2x300 |
| DPT-4C-ext_ITS | 730,391 | C-ext | 2x300 |
| DPT-4C-int_ITS | 597,298 | C-int | 2x300 |
| DPT-5A-ext_ITS | 576,839 | A-ext | 2x300 |
| DPT-5A-int_ITS | 581,057 | A-int | 2x300 |
| DPT-5B-ext_ITS | 629,584 | B-ext | 2x300 |
| DPT-5B-int_ITS | 608,635 | B-int | 2x300 |
| DPT-5C-ext_ITS | 587,116 | C-ext | 2x300 |
| DPT-5C-int_ITS | 625,620 | C-int | 2x300 |
| MC-DPT-4_ITS | 538,626 | M | 2x300 |
| MC-DPT-5_ITS | 665,607 | M | 2x300 |

**Quality analysis of the reads**

Quality analyses were performed over reads using FastQC1 (v0.11.9). S1 Fig 2 shows a representative boxplot of the quality of original reads for 16S (upper panels) and ITS amplicons (lower panels), both forward (usually with better quality, left panels) and reverse sequences (worse quality, right panels).

**S1 Fig 2. Representative quality per base boxplot of reads from sample DPT-1A-ext**

**Primer trimming and joining of paired reads.**

For both amplicons, the first step is trimming the primers from both reads with cutadapt2 <https://cutadapt.readthedocs.io/en/stable/>. Then, quality control is performed again.

For 16S amplicon, after trimming the primers, forward and reverse reads are joined by their overlapping 3’ ends to generate a single longer read with the software pandaseq3 <https://github.com/neufeld/pandaseq>

**S1 Table 4 Primer trimming and joining of paired reads.**

| Step | 16S – Read# | 16S - % original reads | ITS – Read# | ITS - % of original reads |
| --- | --- | --- | --- | --- |
| trimmed reads | 162,187 - 406,103 | 100 | 40,167-730,391 | 100 |
| Joined sequences: merged reads | 160,814–401,589 | 85.4 – 99.15 | * See qiime2 analysis | * See qiime2 analysis |

**Qiime2 analysis.**

For the metagenomics analysis we have used Qiime2 software4. It is an open-source bioinformatics package for performing microbiome analysis from raw DNA sequencing data. Qiime2 is designed to take users from raw sequencing data generated on the Illumina or other platforms through publication of quality graphics and statistics. This includes demultiplexing and quality filtering, OTU picking, taxonomic assignment, phylogenetic reconstruction, diversity analyses and visualizations. Qiime has been applied to studies based on billions of sequences from tens of thousands of samples.

The non-chimeric sequences will be used for the rest of the analysis When looking into negative control values, we can conclude that 16S sequences have contamination, while ITS sequences are clean of contaminant sequences after filtering. We maintained the 16S negative control sample throughout the qiime2 analysis, while it was removed and the data imported again for ITS amplicon, where this sample was not informative.

The sequences of all samples are grouped to define the “amplicon sequence variants” (ASVs) which are the equivalent of 100% Operational Taxonomic Units (OTUs) and will be defined that way from now on. These ASVs are of higher resolution than the Qiime1 (previous software version) default of 97% OTUs and with higher quality as quality control steps are more implemented.

**S1 Table 5. Quality control summary (minimum - maximum ranges)**

| Step | 16S sequence number  (%) | ITS sequence number  (%) |
| --- | --- | --- |
| Input | 372,370 (c-)  160,814 – 401,589 | 97,235 (c-)  105,998 – 730,391 |
|  | 355,370 95.43% (c-) | 40,628 41.78% (c-) |
| Filtered | 145,592 – 355,370 | 80,283 – 695,021 |
|  | (82.71 – 95.82) | (58.63 – 97.76) |
| Denoised | 355,050 (c-)  145,412 – 346,590 | 40,599 (c-)  80,207 – 694,848 |
|  |  | 283 0.29% (c-) |
| Merged | * Sequences previously merged | 72,095 – 691,468 |
|  |  | (50.32 – 97.44) |
|  | 354,730 95.26% (c-) | 283 0.29 (c-) |
| Non-chimeric | 144,712 – 326,881 | 72,095 – 686,946 |
|  | (61.99 – 95.55) | (50.32 – 97.29) |

Finally, there was a total of 2,195 ASVs for 16S and 917 ASVs for ITS. The raw data were deposited in the European Nucleotide Archive (accession number: PRJEB80324).

Regarding chloroplast and mitochondria barcode sequencing contamination in 16S, a filtering step has been applied to remove chloroplast and mitochondria sequences, although the number of sequences assigned to them was quite low (Table 6), with maximum values of 0.6% (mitochondria) and 3.5% (chloroplast).

Regarding potential mite ITS contamination, the UNITE database is specifically curated for fungal ITS sequences and does not comprehensively represent metazoan taxa. Therefore, putative mite-derived reads would most likely remain classified as “unassigned” rather than being explicitly annotated as Acari or other metazoan groups. To evaluate this possibility, we examined the proportion of unassigned ASVs across samples. Most samples showed low and biologically reasonable proportions of unassigned sequences (maximum 13.6%), which is expected in ITS datasets because reference databases such as UNITE do not fully capture the entire environmental fungal diversity. These values are summarized in Table 7.

Sample (DPT-3B-int) showed an unusually high proportion of unassigned sequences (~95%), suggesting potential non-target amplification or poor taxonomic resolution. Therefore, this sample was removed for downstream ITS analyses.
